# Conductivity Extraction Using a 180 GHz Quasi-Optical Resonator for Conductive Thin Film Deposited on Conductive Substrate

**DOI:** 10.3390/ma13225260

**Published:** 2020-11-20

**Authors:** Ming Ye, Xiao-Long Zhao, Wei-Da Li, Yu Zhou, Jia-Yi Chen, Yong-Ning He

**Affiliations:** 1 Faculty of Electronic and Information Engineering, Xi’an Jiaotong University, Xi’an 710049, China; zhaoxiaolong@mail.xjtu.edu.cn (X.-L.Z.); boysid@stu.xjtu.edu.cn (W.-D.L.); zy929585@stu.xjtu.edu.cn (Y.Z.); chenjiayi18@stu.xjtu.edu.cn (J.-Y.C.); yongning@mail.xjtu.edu.cn (Y.-N.H.); 2 State Key Laboratory of Millimeter Wave, Nanjing 210096, China

**Keywords:** microwave effective conductivity, conductive thin film, noncontact, substrate

## Abstract

Measurement of electrical conductivity of conductive thin film deposited on a conductive substrate is important and challenging. An effective conductivity model was constructed for a bilayer structure to extract thin film conductivity from the measured Q-factor of a quasi-optical resonator. As a demonstration, aluminium films with thickness of 100 nm were evaporated on four silicon wafers whose conductivity ranges from ~10^1^ to ~10^5^ S/m (thus, the proposed method can be verified for a substrate with a wide range of conductivity). Measurement results at ~180 GHz show that average conductivities are 1.66 × 10^7^ S/m (which agrees well with direct current measurements) with 6% standard deviation. The proposed method provides a contactless conductivity evaluation method for conductive thin film deposited on conductive substrate which cannot be achieved by the existing microwave resonant method.

## 1. Introduction

Conductive thin films are widely used in applications such as photodetectors [1,2,3], sensors [4,5,6], microwaves [7,8,9], flexible printed electronics [10,11,12] superconductors [13,14,15] and so on. Depending on their application or fabrication process, thin films may be deposited on either an insulating or conductive substrate [16,17,18,19]. One of the most important properties for conductive thin films is electrical conductivity. Since thin film’s conductivity usually depends on the deposition process, film thickness and substrate properties [20,21,22], accurate and convenient conductivity measurement is indispensable.

The four-point probe method is widely used to characterize conductivity [23,24,25]. In this method, it is usually assumed that thin film is deposited on an insulating substrate. However, conductive thin film may be deposited on a conductive substrate, such as copper, carbon fabric, doped silicon [26,27,28] etc. For these cases, the standard four-point probe method may exhibit high measurement error. Some details are included in Supplement Material. A parallel circuit model was proposed to evaluate a coating’s conductivity of bilayer structures using a four-point probe method [29] and some of its details and potential limitation are presented in the Supplementary Materials. Other potential challenges of the standard four-point probe method include: it requires good contact between the probe and thin film (this contact may cause contamination or even damage and, for some special materials, ohmic contact may be hard to form between the probe and sample); it usually measures conductivity under direct current condition (with the development of 5G technology and beyond, thin film characterization at microwave/millimeter-wave/terahertz waves is becoming indispensable). These shortages may limit its application in new materials research and development in the laboratory or in industrial mass production (e.g., process monitoring in an integrated circuit foundry).

Contactless conductivity measurement methods have been reported. Among them, the eddy current method [30,31,32,33] and microwave/terahertz method [34,35,36,37,38,39,40,41,42,43] are especially interesting. Compared with the microwave/terahertz method, the eddy current method usually works at lower frequency, e.g., kHz or MHz. Thus, it is usually suitable for evaluation of a coating with thickness of several microns or bulk conductors. To evaluate thin film coating, due to smaller skin depth, the microwave/terahertz method is preferred. There are two categories of microwave/terahertz method: the transmission/reflection method [34,37,38] and the resonant method [35,36,39,42]. Due to its high sensitivity, the resonant method is frequently used. Since skin depth is inverse proportional to working frequency, it is straightforward, to ensure that the substrate’s effect can be neglected, for very high frequency (e.g., THz band) of electromagnetic waves to be used. For example, suppose conductivity is $3\times 10^{7}$ S/m, skin depth is ~900 nm at 10 GHz and ~90 nm at 1000 GHz. If five skin depths are required to neglect a conductive substrate’s effect, then 100 nm thin film characterization requires the working frequency be at least ~20 THz. Therefore, it is very challenging to evaluate thin films using existing microwave methods when a conductive substrate presents. Furthermore, a network analyzer working in the THz band is expensive and may be still unavailable to many laboratories/companies. Thus, reported works on thin film characterization using microwave/terahertz are mainly focused on thin films with insulating substrates while a few of them deal with a conductive substrate [44,45,46,47].

Here, based on an effective conductivity model of a bilayer structure, we used a quasi-optical resonator to measure a thin film’s conductivity when its substrate is conductive. As far as we know, existing resonant methods cannot be used for this purpose. Compared with existing terahertz methods, our proposed method is expected to be feasible at frequency below THz and thus is potentially more accessible to laboratories/companies involved in new material research and development. With the advantage of the proposed model, a thin film’s thickness can be comparable to or even smaller than the corresponding skin depth. To sum up, due to the quasi-optical resonator, the proposed method is applicable in a wide frequency band (e.g., from tens of GHz to several THz) and it is contactless. These features make the proposed method very flexible and accessible, which will benefit material-related research and development.

## 2. Theory of Measurement Method

**Figure 1:**
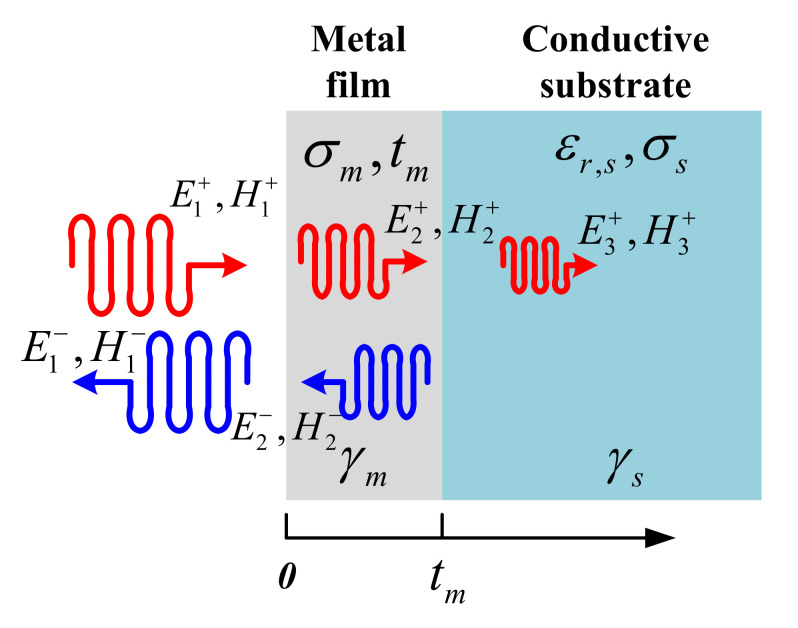
Electromagnetic model of a bilayer structure showing wave transmission/reflection.

The electromagnetic model of transmission/reflection of microwave in the “metallic thin film/conductive substrate” bilayer structure is shown in Figure 1. Here, we consider plane wave. When a microwave is incident onto the surface of thin film from air, due to impedance mismatch, most of the microwave will be reflected back while others will penetrate into the thin film. This penetrated microwave energy continues to propagate toward the conductive substrate while it experiences an obvious attenuation in the thin film. At the “metallic thin film/conductive substrate” interface, both transmission and reflection of microwave energy occurs. Finally, as shown in Figure 1, there are forward and backward electromagnetic waves in thin films and air. Here, it was assumed that all of the microwave energy penetrated into the conductive substrate will be dissipated in it and, thus, no reflected wave exists in the substrate. In Figure 1, E and H represents electric and magnetic fields, respectively. Superscripts “+” and “−” represent forward and backward waves, respectively. Subscript 1, 2 and 3 represents air, metallic thin film and conductive substrate region, respectively. $\sigma_{m}$ and $t_{m}$ represents conductivity and thickness of the thin film, respectively. We assume that the considered thin film is continuous and uniform. $\sigma_{s}$ and $\varepsilon_{r,s}$ represents the conductivity and relative dielectric constant of the conductive substrate, respectively.

According to the boundary conditions at the interface of “air/thin film”, we have:

(1) $$\left\{\begin{array}{c} E_{1}^{+}+E_{1}^{-}=E_{2}^{+}+E_{2}^{-}\\ H_{1}^{+}-H_{1}^{-}=H_{2}^{+}-H_{2}^{-} \end{array}\right.$$

Similarly, at the interface of the “thin film/conductive substrate”, we have:

(2) $$\left\{\begin{array}{c} E_{2}^{+}exp\left(-\gamma_{m}t_{m}\right)+E_{2}^{-}exp\left(\gamma_{m}t_{m}\right)=E_{3}^{+}exp\left(-\gamma_{s}t_{m}\right)\\ H_{2}^{+}exp\left(-\gamma_{m}t_{m}\right)-H_{2}^{-}exp\left(\gamma_{m}t_{m}\right)=H_{3}^{+}exp\left(-\gamma_{s}t_{m}\right) \end{array}\right.$$

Here, propagation constant in the thin film is $\gamma_{m}=\left(1+j\right)/\delta_{s,m}$, skin depth in nano-film is $\delta_{s,m}=1/\sqrt{\pi f\mu_{0}\sigma_{m}}$. j is imaginary unit, f is frequency, $\mu_{0}$ is the permeability of the vacuum (consider only non-magnetic thin film here). Propagation constant in substrate is $\gamma_{s}=j2\pi f\sqrt{\mu_{0}\varepsilon_{0}\varepsilon_{r,s}\left(1-j\sigma_{s}/\left(2\pi f\varepsilon_{0}\varepsilon_{r,s}\right)\right)}$, and here, $\varepsilon_{0}$ is permittivity of vacuum. Total reflection coefficient of the bilayer structure can be obtained by combining Equations (1) and (2):

(3) $$\Gamma_{bilayer}=\frac{E_{1}^{-}}{E_{1}^{+}}=\frac{\left(a_{01}+1\right)\eta_{m}-\left(1-a_{01}\right)\eta_{air}}{\left(a_{01}+1\right)\eta_{m}+\left(1-a_{01}\right)\eta_{air}}$$

To obtain Equation (3), magnetic field is related to electric field by intrinsic impedance as H=E/η. Here, $\eta_{m}=\left(1+j\right)\sqrt{\pi f\mu_{0}/\sigma_{m}}$ and $\eta_{air}=377$ Ω is intrinsic impedance for thin film and air, respectively. $a_{01}=\frac{\eta_{s}-\eta_{m}}{\eta_{s}+\eta_{m}}exp\left(-2\gamma_{m}t_{m}\right)$ and intrinsic impedance for substrate is $\eta_{s}=j2\pi f\mu_{0}/\gamma_{s}$. Equation (3) indicates that the total reflection coefficient depends on the electrical properties of both the thin film ($\sigma_{m}$) and substrate ($\sigma_{s}$/$\varepsilon_{r,s}$), the thickness of the thin film $t_{m}$ and the working frequency f.

**Figure 2:**
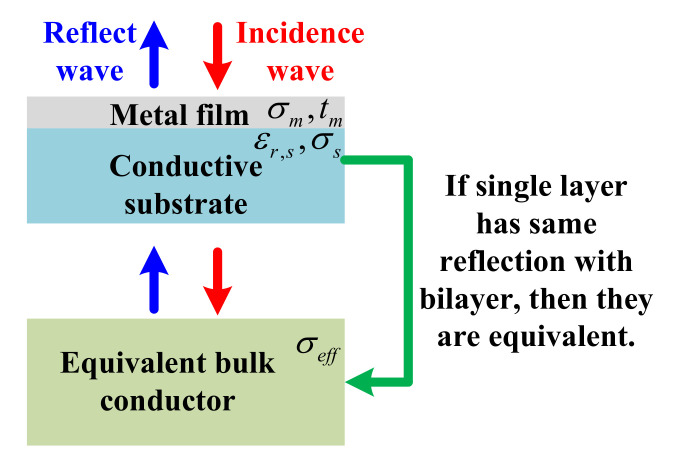
Schematic diagram of effective conductivity definition of a bilayer structure from the reflection point of view.

Effective conductivity for the bilayer structure was defined from the reflection coefficient point of view. As shown in Figure 2, if a semi-infinite bulk conductor with conductivity $\sigma_{eff}$ shows an equal reflection coefficient with the bilayer, we define the bilayer’s effective conductivity as $\sigma_{eff}$. For a bulk conductor with conductivity of $\sigma_{eff}$, its reflection coefficient is [48]:

(4) $$\Gamma_{single-layer}=\frac{\eta_{eff}-\eta_{air}}{\eta_{eff}+\eta_{air}}$$

Here, $\eta_{eff}=\left(1+j\right)\sqrt{\pi f\mu_{0}/\sigma_{eff}}$. By combining Equations (3) and (4), one can relate conductivity of the thin film $\sigma_{m}$ with the effective conductivity of a single layer $\sigma_{eff}$. Therefore, if $\sigma_{eff}$ can be measured, then one can obtain $\sigma_{m}$.

Several kinds of surface resistance $R_{s}$ evaluation method have been described in literatures [49,50,51,52,53] to obtain $\sigma_{eff}$. Among them, the quasi-optical resonator has been widely used and this method is compatible with the theoretical model presented above. Effective conductivity is related with $R_{s}$ through:

(5) $$\sigma_{eff}=\pi f\mu_{0}/R_{s}^{2}$$

**Figure 3:**
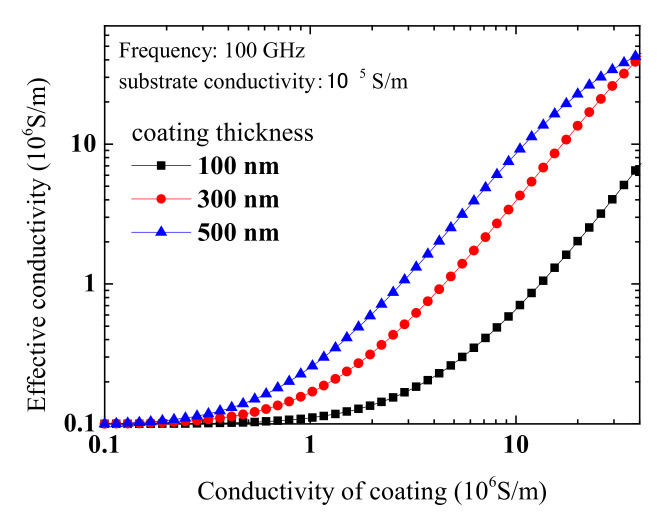
Calculated dependence of effective conductivity of bilayer structure on conductivity of the thin film.

In Figure 3, as a demonstration, the dependence of the effective conductivity on the conductivity of thin film is numerically calculated using Equations (3)–(5) at a frequency of 100 GHz and substrate’s conductivity of $10^{5}$ S/m. Calculations at other frequencies show similar results. The considered thicknesses of thin films includes 100, 300 and 500 nm. This shows that the effective conductivity increases with increasing conductivity of thin film. So, if the thickness of thin film and conductivity of substrate are both known, one can extract the thin film’s conductivity from measured effective conductivity. This is the basic idea in the measurement method proposed here.

## 3. Experiment and Discussion

**Figure 4:**
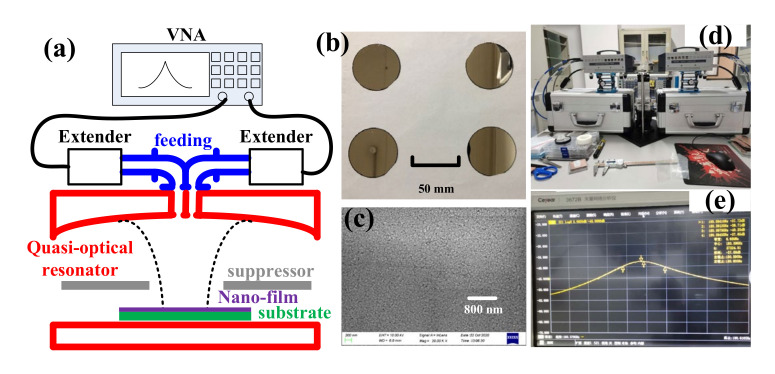
(**a**) Schematic diagram of measurement system; (**b**) photo of used samples; (**c**) scanning electron microscope photo of deposited aluminium thin film; (**d**) photo of used quasi-optical resonator; (**e**) measured resonant curve at ~180 GHz.

In experimental verifications, as a proof of concept, four silicon wafers with various doping levels were used as conductive substrates. The reasons for using doped silicon as conductive substrates included: first, by controlling the doping level, these wafers have their conductivity ranging from ~20 S/m to $10^{5}$ S/m; second, it is expected that silicon wafers are ideally flat and thus exclude a possible surface roughness effect on high-frequency conductivity. All of these wafers are of diameter 50.8 mm and thickness 0.4 mm. Conductivity of these substrates was measured using the four-point probe method (RTS-8, 4p robes tech, Inc. China) as listed in the first column of Table 1. By using electron beam evaporation (Ohmiker-50BR, M&R Nano Technology Co. Ltd, Taoyuan, Taiwan), aluminium thin film was deposited on silicon with deposition rate ~0.2 nm/s and the total thickness is 100 nm. To ensure consistency of conductivity, all of the four samples were deposited in one batch. A photo of the samples is included in Figure 4b. From the SEM (scanning electron microscope) photo shown in Figure 4c, it can be seen that the deposited Al thin film is continuous and flat.

Four-point probe method was used to evaluate sheet resistance of these silicon wafers deposited with aluminium thin film and the results obtained are listed in the second column of Table 1. The sheet resistance of the first three samples are similar (~600 mΩ/sq) while the last sample has an obviously different sheet resistance, namely, 285 mΩ/sq. Conductivity of thin films $\sigma_{m}$ calculated from the second column value $R_{Si/Al}$ using $\sigma_{m}=1/\left(R_{Si/Al}t_{m}\right)$ is shown in the third column of Table 1. Here, $R_{Si/Al}$ is the measured sheet resistance of silicon deposited with Al thin film. It can be seen that the conductivity of the last sample ($3.5\times 10^{7}$ S/m) is about twice that of the first three samples ($1.6\times 10^{7}$ S/m). Considering the fact that these samples were deposited in one batch on similar polished silicon substrates, one can expect that their conductivities should have little difference. Thus, it can be concluded that, for the last sample, the substrate’s good conductivity has an obvious effect on aluminium thin film conductivity evaluation. It is not accurate to evaluate thin film’s conductivity using a standard four-point probe method when thin film’s substrate has relatively good conductivity. However, for the first three samples, since the substrate has relatively low conductivity, the four-point probe measurement results are trustworthy. Therefore, it is expected that the conductivity of the deposited aluminium thin film is $1.66\times 10^{7}$ S/m (averaged value for the first three samples obtained with a four-point probe). This will be used as a reference value later.

Finally, as a proof of concept and due to the availability of setups, a quasi-optical resonator working at ~180 GHz was adopted to measure the conductivity of the thin film. The resonator is composed of a spherical and plane mirror. The curvature radius of the spherical mirror is 42 mm and the cavity length is 26 mm. As shown in Figure 4a, the sample under test is used as the plane mirror (the photo of the used quasi-optical resonator is shown in Figure 4d and a typical resonant curve obtained from VNA (Vector Network Analyzer) is shown in Figure 4e). The working mode is TEM_0030_. Suppressor made by copper clad laminate was used to supress higher order modes. A vector network analyzer (3672E, China Electronics Technology Instruments Co., Ltd, Qingdao, China) and millimeter-wave extenders (3643S, China Electronics Technology Instruments Co., Ltd, Qingdao, China) were used for Q-factor measurement. As described in [47], dependence of the resonator’s unloaded Q factor $Q_{0,sample}$ on the surface resistance $R_{s,sample}$ of a sample can be written as:

(6) $$R_{s,sample}=AQ_{0,sample}^{-1}+B$$

Here, A and B are constants determined by the geometrical size of the cavity and its surface treatments (e.g., polishing or silver plating is expected to increase the Q factor). In our experiment, two calibration samples with known conductivity were used to obtain these two constants. To ensure repeatability of measurement data, all of the samples were measured four times. Details of the measurement are included in the Supplementary Materials. After obtaining surface resistance from the measured unloaded Q-factor, one can use Equation (5) to calculate the effective conductivity as listed in the fourth column of Table 1. The effective conductivity is independent of the substrate’s conductivity and they are about one order of magnitude smaller than the values shown in the third column. Using the effective conductivity model described above, the thin film’s conductivities were extracted and they are shown in the fifth column of Table 1. Extracted thin film conductivities are consistent and reasonable: averaged conductivity is $1.66\times 10^{7}$ S/m (close to the conductivity from the four-point probe mentioned above) and standard deviation is $0.98\times 10^{6}$ S/m (indicating 6% variation). It should be noted that, through theoretical analysis, the proposed method is expected to be feasible for the low microwave frequency band such as below 30 GHz, although we demonstrated this with a ~180 GHz resonator.

**Figure 5:**
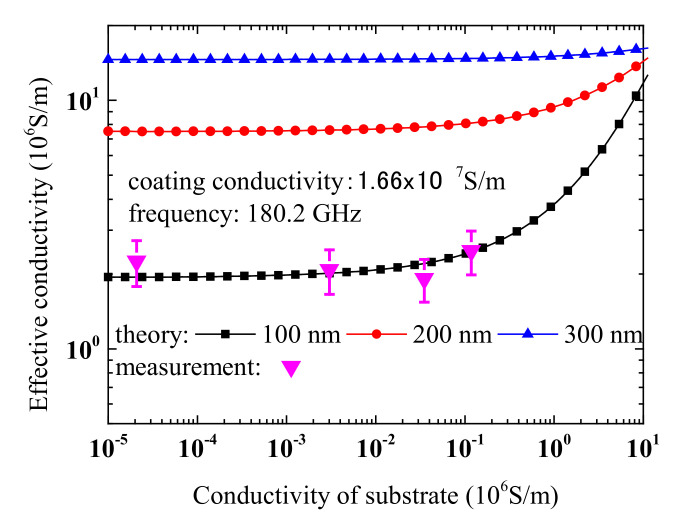
Calculated dependence of effective conductivity of bilayer structure on conductivity of substrate. Measurement results are also presented for comparison.

In Figure 5, we show calculation results on the dependence of effective conductivity on the substrate’s conductivity. Measurement results are also included for comparison. Theoretical predictions agree well with experiments. This indicates that both our theory and measurements are correct. It should be noted that, here, we assume that the relative dielectric constant of silicon is a constant, namely, 11.9. In fact, dielectric constant may change with doping level [54,55]. However, our calculations show that the relative dielectric constant has little effect on extracted conductivity for cases discussed here. Also, we calculated the potential effect of oxide film on aluminium thin film and result shows that it has little effect on conductivity extraction, even considering the fact that the permittivity of alumina thin film may be different with bulk material [56]. Some details are included in Supplement Material.

Uncertainty analysis shows that the main contribution of measurement uncertainty is Q factor uncertainty and the total uncertainty was estimated to be ~7%. This conclusion agrees with reported results such as [42]. Details of uncertainty analysis are provided in the Supplementary Materials. It may be possible to improve Q factor measurement uncertainty using some other reported method [57]. By polishing the substrate, it is expected that the used samples are ideally flat. However, as presented in [58], surface roughness may affect conductivity at high frequency. Future research may take surface roughness effect into consideration. Here, we only consider the case that a substrate’s conductivity is lower than its coating and the inverse case will be studied in the near future.

## 4. Conclusions

In conclusion, we proposed a contactless method for evaluating the conductivity of conductive thin film deposited on a conductive substrate. Using the developed effective conductivity theory of a bilayer structure and measured effective conductivity of doped silicon wafers deposited with 100 nm thick aluminium thin film from a quasi-optical resonator working at ~180 GHz, we demonstrated both theoretically and experimentally that the proposed method is feasible. The estimated conductivity of deposited aluminium thin film is ~$1.66\times 10^{7}$ S/m. This work provides an alternative for characterization of conductive thin film deposited on a conductive substrate for both scientists and engineers. Potential future work may include: (1) verification of the proposed method with other thin films (including other materials and thicknesses, such as thickness below 100 nm) and substrates; (2) verification of the proposed method with another microwave resonant cavity working at lower frequency such as below 30 GHz; (3) extension of the proposed method for evaluating electrical parameters of both the thin film and substrate or multilayer structures.

## Figures and Tables

**Table 1:** Measurement results from four point probe and microwave method.

Silicon’s Conductivity (S/m)	Si/Al Sheet Resistance (mΩ/sq)	Al film’s Conductivity $(10^{6}S/m{)}^{1}$	Effective Conductivity $\left(10^{6}S/m\right)$	Al film’s Conductivity $(10^{6}S/m{)}^{2}$
20.8	616	16.2	2.25	17.7
3.03 × 10^3^	581	17.2	2.08	16.7
3.50 × 10^4^	606	16.5	1.92	15.3
1.18 × 10^5^	285	35.1	2.48	16.6

^1^ Four-point probe method (conductivity = (1/(Si/Al sheet resistance∗Al thickness))); ^2^ Microwave method.

## Supplementary Materials

The following are available online at https://www.mdpi.com/1996-1944/13/22/5260/s1, Figure S1: Principle of four point probe method; Figure S2: Typical electromagnetic field distribution of one port feed quasi optical resonator; Figure S3: SEM photo of deposited aluminium film. Table S1: Four-point probe measurement results; Table S2: Measurement results of the two calibration samples; Table S3: Measurement results of the four aluminium thin film samples; Table S4: Measurement results from four point probe and microwave method. Some MATLAB codes are also included.
