# Supplementary material for: Conductivity Extraction Using a 180 GHz Quasi-Optical Resonator for Conductive Thin Film Deposited on Conductive Substrate
Source: Materials (Basel). 2020 Nov 20;13(22):5260. doi: 10.3390/ma13225260 (PMC7699854; doi:10.3390/ma13225260)
Supplement: Supplementary file 1 [file materials-13-05260-s001.pdf]

materials

Supplementary Materials: Conductivity Extraction Using a 180 GHz Quasi-Optical Resonator for Conductive Thin Film Deposited on Conductive Substrate

Ming Ye [1,2,*], Xiao-Long Zhao [1], Wei-Da Li [1], Yu Zhou [1], Jia-Yi Chen [1] and Yong-Ning He [1]

[1] Faculty of Electronic and Information Engineering, Xi'an Jiaotong University, 710049 Xi'an, China; zhaoxiaolong@mail.xjtu.edu.cn (X.-L.Z.); boysid@stu.xjtu.edu.cn (W.-D.L.); zy929585@stu.xjtu.edu.cn (Y.Z.); chenjiayi18@stu.xjtu.edu.cn (J.-Y.C.); yongning@mail.xjtu.edu.cn (Y.-N.H.)
[2] State Key Laboratory of Millimeter wave, 210096 Nanjing, China
* Correspondence: yeming057@mail.xjtu.edu.cn;

1. Four-Point Probe

The principle of the four-point probe method is shown as follows: probes 1 and 4 are used to input and output the excitation current, respectively; probes 2 and 3 are used to measure voltage drop inside the sample caused by the excitation current.

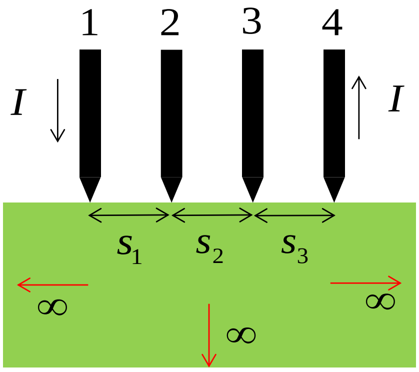

Figure S1. Principle of four-point probe method.

If the sample is semi-infinite, as shown above, the voltage drop should be (see "Dieter K. Schroder, Semiconductor material and device characterization, third edition, chapter 1, John Wiley and Sons, Inc., publication"):

$$V_{23} = \frac{I\rho}{2\pi}\left(\frac{1}{s_1} - \frac{1}{s_2 + s_3} - \frac{1}{s_1 + s_2} + \frac{1}{s_3}\right) \tag{1}$$

Here, V_{23} is voltage drop between probe 2 and probe 3, I is the excitation current, ρ is resistivity of material under test, S_1, S_2, S_3 is distance between adjacent probes as shown in Figure S1. Therefore, if one knows current and probe distance, then resistivity can be calculated from measured voltage drop.

For a conductive nano-film deposited on insulating material, a popular formula for calculating sheet resistance of nano-film is:

$$R_S = \frac{\rho}{t} = 4.532\frac{V_{23}}{I} \tag{2}$$

After obtaining sheet resistance R_s one can obtain resistivity ρ if thickness t can be determined by some other method (e.g., scanning electron microscope or stylus profiler). This method has been widely used in semiconductor laboratories and industry.

However, in this work, we focus on a nano-film deposited on a conductive substrate. In particular, we want to discuss the case that a substrate has good conductivity or has a conductivity close to the nano-film itself (here, we focus on the case that the conductivity of the nano-film is higher than its substrate). Deposition on a conductive substrate may be encountered in, to name a few, a metallic nano-film deposited on doped silicon, other semiconductor materials, other bulk conductive materials, alloys, or epitaxial film on semiconductor. For this case, using the standard four-point probe method mentioned above to evaluate the conductivity of a nano-film may be inaccurate or even incorrect. It can be explained as follows: if the substrate is conductive to some degree, a part of the excitation current input into the sample will flow in the substrate besides the nano-film under evaluation. Thus, the measured conductivity is in fact contributed to by both the nano-film and its substrate. In other words, the measured conductivity of the nano-film would be higher than its real value. Since both the coating and substrate have contributed to electrical conductivity, it is in fact a two-layer structure or bilayer structure.

Some papers have been published to tackle the conductivity measurement of such a bilayer structure using the four-point probe method. If a potential reader is interested in the four-point probe method developed for a two-layer structure, he or she may refer "Y. Y. Chen and J. Y. Juang, Meas. Sci. Technol. 27:074006(2016). Finite element analysis and equivalent parallel-resistance model for conductive multilayer thin films". (One can also refer to "M. A. C. S. Brown and E. Jakeman, Theory of the four-point probe technique as applied to the measurement of the conductivity of thin layers on conducting substrates, 1966, Br. J. Appl. Phys. 17 1143", "Mardochee Reveil, Victoria C. Sorg, Emily R. Cheng, Taha Ezzyat, Paulette Clancy, and Michael O. Thompson, Finite element and analytical solutions for van der Pauw and four-point probe correction factors when multiple non-ideal measurement conditions coexist, Review of Scientific Instruments 88, 094704 (2017); doi: 10.1063/1.5001830")

In Y. Y. Chen and J. Y. Juang's paper, a measurement method is introduced to measure the resistivity of multilayer structures. Using the parallel circuit model presented in this paper, sheet resistance of nano-film $R_{s,coating}$, substrate $R_{s,substrate}$ and the bilayer structure $R_{s,eff}$ can be written as:

$$\frac{1}{R_{s,coating}}+\frac{1}{R_{s,substrate}}=\frac{1}{R_{s,eff}} \tag{3}$$

There are two major assumptions in this model: first, there is no additional interface resistance between the two layers; second, voltage potential drop across the top layer can be neglected. Considering the fact that $R_s = 1/(\sigma t)$, (t is thickness), one can obtain:

$$\sigma_{coating}=\frac{\frac{1}{R_{s,eff}}-\frac{1}{R_{s,substrate}}}{t_{coating}} \tag{4}$$

Therefore, if we know sheet resistance of the bilayer structure and substrate as well as the coating's thickness, we can calculate the coating's conductivity using the formula above.

From a mathematical point of view, if $R_{s,eff} > R_{s,substrate}$, then the calculated coating's conductivity is a minus value. In our previous manuscript, we have shown the following data in Table S1. It can be seen that for the last two samples, $R_{s,eff} > R_{s,substrate}$. Thus, one will get a negative conductivity for the coating. This case may occur when the two assumptions are invalid. We have also conducted some finite element simulations and we found that the parallel circuit

model mentioned above may be also suitable for our samples (simulated potential drop agrees well with prediction values from the parallel circuit model). This indicates that the second assumption "potential drop across thickness can be neglected" may be correct. Therefore, we guess it is because of the interface resistance that makes the parallel circuit model fail to explain our measurement results. However, if one wants to prove this point, more effort is needed (for example, use of a special method to measure contact resistance or measure the current-voltage characteristics). Since the focus of our work is introducing a contactless measurement method, we do not try to solve this contact resistance problem at the moment. It may be retained for future work.

Table S1. Four-point probe measurement results.

Silicon's Conductivity (S/m)	Si sheet Resistance （mΩ/sq）	Si/Al sheet Resistance （mΩ/sq）
20.8	$1.20*10^5$	616
$3.03*10^3$	825	581
$3.50*10^4$	71.4	606
$1.18*10^5$	21.3	285

2. Quasi-Optical Resonator Measurement

The quasi-optical resonator has been studied for decades. Many people use this kind of resonator to characterize a material's electrical properties, such as relative dielectric constant or loss tangent of low loss dielectric material, surface resistance of a superconductor, surface conductivity of bulk conductors. Advantages of this kind of resonator include: it is relatively easy to work with at a higher frequency band like millimeter wave or terahertz band with high Q factor; a quasi-optical resonator has an open structure and thus it is convenient for contactless measurement.

In this work, we use a semi-spherical quasi-optical resonator to characterize surface conductivity, which has been reported in existing publications. As suggested in literature, we use TEM_{00q} mode whose resonant frequency can be described as:

$$f_{00q} = \frac{c}{2D}\left(q + 1 + \frac{1}{\pi}\arctan\left(\sqrt{\frac{D}{R-D}}\right)\right) \quad (5)$$

Here, c is light speed in vacuum, D is cavity length, q is mode number, R is curvature radius of the spherical mirror. For this mode, most of the electromagnetic energy is confined close to the center of the cavity. It is possible to feed the resonator with either two ports or one port. In this work, we use two ports to feed the cavity. Thus, S_{21} is measured to obtain Q factor. A typical electromagnetic field distribution obtained from simulation is shown in Figure S2.

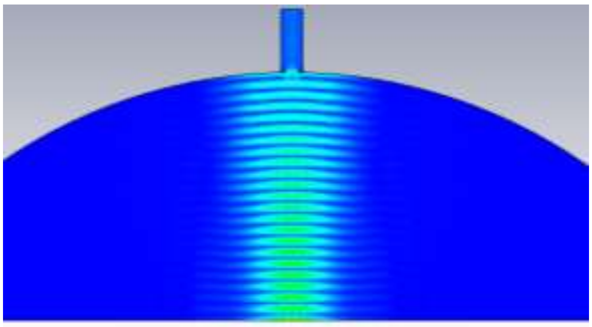

Figure S2. Typical electromagnetic field distribution of one-port feed quasi-optical resonator.

Before we measure the sample under test, we first calibrate our resonator. Two samples with known conductivity were used: one polished aluminium alloy and one polished doped silicon. These two samples are denoted as calibration samples. Polishing treatment ensures that the potential effect of surface roughness on high-frequency conductivity can be minimized For the aluminium alloy calibration sample, its conductivity was measured using the eddy current method which shows 25.8 MS/m. For the silicon calibration sample, its conductivity was measured using the four-point probe method which shows 0.111 MS/m. To observe repeatability, four runs were conducted. For each run of the test, the resonator was calibrated before measuring the samples under test (aluminium coated silicon wafers). Typical measurement results of these two calibration sample are shown as follows.

Table S2. Measurement results of the two calibration samples.

Sample	Resonant Frequency (GHz)	Loaded Q-Factor	Insertion Loss at f_0(dB)	Unloaded Q-Factor
Aluminium Alloy	180.61	28486	37.89	28854
Doped Silicon	180.73	5427	49.03	5446

Now, we want to show how to derive calibration coefficients. For calibration sample 1, sample 2 and the sample under test, we have:

$$Q_{total,cal1}^{-1} = Q_{sphere}^{-1} + Q_{cal1}^{-1} = Q_{sphere}^{-1} + c_{flat} R_{s,cal1} \tag{6}$$

$$Q_{total,cal2}^{-1} = Q_{sphere}^{-1} + Q_{cal2}^{-1} = Q_{sphere}^{-1} + c_{flat} R_{s,cal2} \tag{7}$$

$$Q_{total,sample}^{-1} = Q_{sphere}^{-1} + Q_{sample}^{-1} = Q_{sphere}^{-1} + c_{flat} R_{s,sample} \tag{8}$$

Here, $Q_{total,cal1}$, $Q_{total,cal2}$, $Q_{total,sample}$ is total unloaded Q factor of resonator when loaded with calibration samples 1, 2 and the sample under test, respectively . Q_{sphere}, Q_{cal1}, Q_{cal2}, Q_{sample} is unloaded Q factor contributed by spherical mirror, calibration sample 1, 2 and sample under test, respectively. It should be noted that inverse of Q factor for plane mirror is proportional to surface resistance with a proportionality factor c_{flat}. Thus, we have $Q_{cal1}^{-1} = c_{flat} R_{s,cal1}$, $Q_{cal2}^{-1} = c_{flat} R_{s,cal2}$, $Q_{sample}^{-1} = c_{flat} R_{s,sample}$ .By combining these three equations, we have:

$$\begin{aligned} R_{s,sample} &= \frac{R_{s,cal1} Q_{total,cal2}^{-1} - R_{s,cal2} Q_{total,cal1}^{-1}}{Q_{total,cal2}^{-1} - Q_{total,cal1}^{-1}} + \frac{-R_{s,cal1} + R_{s,cal2}}{Q_{total,cal2}^{-1} - Q_{total,cal1}^{-1}} Q_{total,sample}^{-1} \\ &= A Q_{total,sample}^{-1} + B \\ &\Rightarrow A = \frac{-R_{s,cal1} + R_{s,cal2}}{Q_{total,cal2}^{-1} - Q_{total,cal1}^{-1}}, B = \frac{R_{s,cal1} Q_{total,cal2}^{-1} - R_{s,cal2} Q_{total,cal1}^{-1}}{Q_{total,cal2}^{-1} - Q_{total,cal1}^{-1}} \end{aligned} \tag{9}$$

With the measurement results presented in Table S2, we then obtain: A is equal to 15,896 Ω and B is equal to –0.38467 Ω. Thus, using these two coefficients, one can calculate surface resistance of sample under test. Assuming the measured sample is a single-layer bulk conductor, we can obtain the sample's effective conductivity from measured surface resistance. Finally, using the derived effective conductivity of a bilayer structure, we can extract a nano-film's conductivity. A group of measurement results of the quasi-optical resonator when loaded with the sample under test are given in Table S3. In Table S4, we present measurement results of all of the four test runs. It can be

seen that the obtained results are satisfactorily repeatable. Uncertainty analysis given later will be of help to understand the repeatability. In our manuscript, averaged values are presented in Table 1.

Table S3. Measurement results of the four aluminium nano-film samples.

Silicon's Conductivity (S/m)	Resonant Frequency (GHz)	Loaded Q-Factor	Insertion Loss at f_0 (dB)	Unloaded Q-Factor
20.8	180.59	15126	41.22	15259
3.03×10^3	180.54	14824	41.67	14947
3.50×10^4	180.62	14679	41.98	14797
1.18×10^5	180.38	15662	41.51	15795

Table S4. Measurement results from four-point probe and microwave method.

	Effective Conductivity（MS/m）				Al film's Conductivity（MS/m）			
	Test 1	Test 2	Test 3	Test 4	Test 1	Test 2	Test 3	Test 4
20.8	2.43	1.65	2.16	2.77	18.4	15.1	17.4	19.7
3.03×10^3	2.38	1.55	1.93	2.46	18.0	14.4	16.2	18.3
3.50×10^4	2.25	1.50	1.70	2.21	16.7	13.4	14.4	16.6
1.18×10^5	2.77	1.84	2.35	2.96	17.7	14.0	16.1	18.4

MATLAB code for extracting coating's conductivity.

The following MATLAB code was written to extract the coating's conductivity. It may be of help for potential readers.

```
% Written by Ming Ye @ Xi'an Jiaotong University
% this code is used to extract the coating's conductivity from measurements
% surface resistance or effective conductivity
% international standard unit is used in this code

clc;clear all;close all;
% vacuum permeability
mu_0=4*pi*1e-7;
% vacuum permittivity
epsilon_0=8.854e-12;

% measured resonant frequency
f_res=180.383e9;
% measured surface resistance
test_Rs=0.622;

% coating thickness
```

```
thick_coat=0.1e-6;
% relative dielectric constant of silicon wafer
epsilon_r_bulk=11.9;
% conductivity of silicon wafer
sigma_bulk=1.176e5;

% angular frequency
omega_res=2*pi*f_res;
% calculated effective conductivity from measured surface resistance
sigma_eff_res_freq=omega_res*mu_0/2/test_Rs^2;

% propagation constant in effective bulk conductor
gamma_eff_bulk=1j.*omega_res.*sqrt(mu_0.*epsilon_0).*sqrt(1-1j.*sigma_eff_res_freq./(omega_res.*
epsilon_0));
% intrinsic impedance in air
eta0=377;
% intrinsic impedance in effective conductor
eta_eff_bulk=1j.*omega_res.*mu_0./gamma_eff_bulk;
% reflection coefficient of effective conductor
R_eff_bulk=(eta_eff_bulk-eta0)./(eta_eff_bulk+eta0);
% reflection coefficient of effective conductor in dB
R_eff_bulk_dB=20.*log10(abs(R_eff_bulk));

%% using sweeping method to extract coating's conductivity
f01=f_res;
sigma_coat_sweep=logspace(log10(0.1e6),log10(30e6),1e4);
S11_dB01test=zeros(1,length(sigma_coat_sweep));
for nn01=1:length(sigma_coat_sweep)
sigma_coat01=sigma_coat_sweep(nn01);
S11_dB01test(nn01)=nsfc_20201004_001(f01,thick_coat,sigma_coat01,sigma_bulk,epsilon_r_bulk);
end
% difference between reflection of effective conductor and bilayer structure
diff=abs(S11_dB01test-R_eff_bulk_dB);
% find out the minimum difference
[n01,n02]=min(diff);
% obtained the coating's conductivity
sigma_coat_estimate=sigma_coat_sweep(n02);

function S11_dB=nsfc_20201004_001(f,thick_coat,sigma_coat,sigma_bulk,epsilon_r_bulk)
epsilon_0=8.854e-12;
mu_0=4*pi*1e-7;
omega=2.*pi.*f;
skin_depth_coat=1./sqrt(pi.*f.*mu_0.*sigma_coat);
```

```
eta0=377;
gamma_coat=(1+1j)./skin_depth_coat;
gamma_bulk=1j.*omega.*sqrt(mu_0.*epsilon_0.*epsilon_r_bulk.*(1-1j.*sigma_bulk./(omega.*epsilon
_0.*epsilon_r_bulk)));
eta_coat=gamma_coat./sigma_coat;
eta_bulk=1j.*omega.*mu_0./gamma_bulk;
a01=exp(-2.*gamma_coat.*thick_coat).*(eta_bulk-eta_coat)./(eta_bulk+eta_coat);
R=((a01+1).*eta_coat-(1-a01).*eta0)./((a01+1).*eta_coat+(1-a01).*eta0);
S11_dB=20.*log10(abs(R));
```

3. Uncertainty Analysis

Measurement uncertainty of our method is related to the following factors: uncertainty of measurement results of calibration samples (including conductivity, resonant frequency, unloaded Q factor), and uncertainty of measurement results of sample under test (including resonant frequency, unloaded Q factor, nano-film's thickness, substrate's conductivity).

To evaluate measurement uncertainty quantitatively, the following data are used (if available, most of these data considered the measurement instruments we used in this work): uncertainty of Q factor measurement is 1%; uncertainty of eddy current measurement is 1%; uncertainty of four point probe measurement is 4%; uncertainty of nano-film thickness measurement is 1%. It should be noted that, as described in literature, uncertainty of resonant frequency measurement is usually secondary and can be neglected.

Overall, in the proposed method, there are two steps to obtain a nano-film's conductivity: first, measure the sample's effective conductivity; second, extract the nano-film's conductivity. Uncertainty contribution from the first step includes: uncertainty of Q factor measurement, uncertainty of eddy current measurement, uncertainty of four-point probe measurement. Uncertainty contribution from the second step includes: uncertainty of four-point probe measurement (substrate's conductivity/permittivity) and uncertainty of nano-film thickness measurement.

For the first contribution, surface resistance of the sample under test is related to calibration samples as follows:

$$R_{s,sample} = \frac{R_{s,cal1}Q^{-1}_{total,cal2} - R_{s,cal2}Q^{-1}_{total,cal1}}{Q^{-1}_{total,cal2} - Q^{-1}_{total,cal1}} + \frac{-R_{s,cal1} + R_{s,cal2}}{Q^{-1}_{total,cal2} - Q^{-1}_{total,cal1}} Q^{-1}_{total,sample} \quad (10)$$

Effective surface conductivity can be represented as:

$$\sigma_{s,sample} = \pi f \mu_0 / R^2_{s,sample} \quad (11)$$

Surface resistance of calibration sample can be represented as:

$$\begin{aligned} R_{s,cal1} &= \sqrt{\pi f \mu_0 / \sigma_{s,cal1}} \\ R_{s,cal2} &= \sqrt{\pi f \mu_0 / \sigma_{s,cal2}} \end{aligned} \quad (12)$$

With these formulations as well as the uncertainty data mentioned above, uncertainty of the first step can be calculated as follows: for each quantity, a perturbation was applied and the obtained perturbed result is compared with the original value. A typical MATLAB code for uncertainty calculation is also attached later. Calculation results show that the total uncertainty for the first step is ~10% and its composition is: four-point probe contributes ~3%, eddy current contributes ~1%, Q factor contributes 6%.

Similarly, we evaluate uncertainty of the second step and results show that the total uncertainty is ~7% and its composition is: four-point probe contributes ~0.3%, effective conductivity contributes ~6%, nano-film thickness contributes 1%.

The following MATLAB code was used for the uncertainty analysis mentioned above.

```
clc;clear all;close all;
mu_0=4*pi*1e-7;
epsilon_0=8.854e-12;
f_low=180.383e9;
omega_low=2*pi*f_low;
test_Rs=0.622;
sigma_eff_low_freq=omega_low*mu_0/2/test_Rs^2;
thick_coat=0.1e-6;
sigma_bulk=1.176e5;
gamma_low=1j.*omega_low.*sqrt(mu_0.*epsilon_0).*sqrt(1-1j.*sigma_eff_low_freq./(omega_low.*e
psilon_0));
eta0=377;
eta_low=1j.*omega_low.*mu_0./gamma_low;
R_low=(eta_low-eta0)./(eta_low+eta0);
R_low_dB=20.*log10(abs(R_low));
epsilon_r_bulk=11.9;
f01=f_low;
sigma_coat_sweep=logspace(log10(0.1e6),log10(30e6),1e4);
for nn01=1:length(sigma_coat_sweep)
sigma_coat01=sigma_coat_sweep(nn01);
S11_dB01test(nn01)=nsfc_20201004_001(f01,thick_coat,sigma_coat01,sigma_bulk,epsilon_r_bulk);
end
diff=abs(S11_dB01test-R_low_dB);
[n01,n02]=min(diff);
sigma_coat_estimate=sigma_coat_sweep(n02);

delta_eff=0.0;
delta_thick=0.01;
delta_sigma=0.0;
mat01=ones(8,3).*-2;
row01=1;
for nn01=-1:1
for nn02=-1:1
for nn03=-1:1
mat01(row01,:)=[nn01 nn02 nn03 ];
row01=row01+1;
end
end
end
for nnn01=1:length(mat01(:,1))
```

```
mat02(nnn01,:)=mat01(nnn01,:).*[delta_eff delta_thick delta_sigma];
sigma_coat_sweep=logspace(log10(0.1e6),log10(30e6),1e4);
for nn01=1:length(sigma_coat_sweep)
sigma_coat01=sigma_coat_sweep(nn01);
S11_dB01test(nn01)=nsfc_20201004_001(f01,thick_coat.*(1+mat02(nnn01,2)),sigma_coat01,sigma_bul
k.*(1+mat02(nnn01,3)),epsilon_r_bulk);
end
gamma_low=1j.*omega_low.*sqrt(mu_0.*epsilon_0).*sqrt(1-1j.*sigma_eff_low_freq.*(1+mat02(nnn0
1,1))./(omega_low.*epsilon_0));
eta0=377;
eta_low=1j.*omega_low.*mu_0./gamma_low;
R_low=(eta_low-eta0)./(eta_low+eta0);
R_low_dB=20.*log10(abs(R_low));
diff=abs(S11_dB01test-R_low_dB);
[~,n02]=min(diff);
sigma_coat_estimate01(nnn01)=sigma_coat_sweep(n02);
end
sigma_low=min(sigma_coat_estimate01);
sigma_high=max(sigma_coat_estimate01);
ratio_low=(sigma_low-sigma_coat_estimate)/sigma_coat_estimate
ratio_high=(sigma_high-sigma_coat_estimate)/sigma_coat_estimate
```

4. Oxide Effect

It is widely recognized that aluminium usually has alumina on its surface due to oxidation. In our experiments, it can be expected that alumina with a thickness of nanometers may be formed on the surface of aluminium nano-film. Therefore, in this case, a bilayer structure (aluminium nano-film on top of silicon substrate) transfers to a tri-layer structure (silicon /aluminium/ alumina). Using the same derivation method with the bilayer structure, we obtained the reflection coefficient for the tri-layer structure:

$$R=\frac{E_1^-}{E_1^+}=-\frac{\left((1+a_{02})-\frac{\eta_{coat,1}}{\eta_0}(1-a_{02})\right)}{\left((1+a_{02})+\frac{\eta_{coat,1}}{\eta_0}(1-a_{02})\right)} \tag{13}$$

where,

$$a_{01}=\frac{\exp\left(-\gamma_{coat,2}t_{coat,2}\right)\left(1-\frac{\eta_{bulk}}{\eta_{coat,2}}\right)}{\exp\left(\gamma_{coat,2}\left(t_{coat,1}+t_{coat,2}\right)\right)\left(1+\frac{\eta_{bulk}}{\eta_{coat,2}}\right)} \tag{14}$$

$$a_{02}=\frac{\exp\left(-2\gamma_{coat,1}t_{coat,1}\right)\left(\left(1-\frac{\eta_{coat,2}}{\eta_{coat,1}}\right)\exp\left(-\gamma_{coat,2}t_{coat,1}\right)+\left(1+\frac{\eta_{coat,2}}{\eta_{coat,1}}\right)a_{01}\right)}{\left(\left(1+\frac{\eta_{coat,2}}{\eta_{coat,1}}\right)\exp\left(-\gamma_{coat,2}t_{coat,1}\right)+\left(1-\frac{\eta_{coat,2}}{\eta_{coat,1}}\right)a_{01}\right)} \tag{15}$$

Here, 'coat,1' represents the top layer (here, it is alumina), 'coat,2' represents the middle layer (here, it is aluminium), and 'bulk' represents the bottom layer (here, it is the silicon wafer).

With the above derived formula, we can estimate the possible effect of alumina on the reflection coefficient (if alumina has some effect on the reflection coefficient, then it is expected that alumina will also has some effect on the extracted coating's conductivity). In our calculations, we set the relative dielectric constant of alumina as 9.9 and its loss tangent as 0.01; we found even a 100 nm (this thickness should be much larger than the thickness of naturally formed alumina) alumina has little effect on an extracted nano-film's conductivity.

5. Substrate's Permittivity

In our work, we have assumed that doping level has little effect on the relative dielectric constant of silicon. However, some published work shows that the relative dielectric constant may change with doping level (for example, see "S. Ristić, A. Prijić, and Z. Prijić. Dependence of Static Dielectric Constant of Silicon on Resistivity at Room Temperature. Serbian Journal of Electrical Engineering, vol.1, no.2, 2004, 237-247", "S. K. Varadan. Estimation of Complex Permittivity of Silicon at 2.45 GHz Microwave Frequency. Master Thesis, Arizona State University, 2014"). Therefore, we tuned the relative dielectric constant of silicon substrate in our calculations to observe its possible effect on extracted conductivity. Results show that, even with a relative dielectric constant of 50, the extracted nano-film's conductivity is the same as the case when relative dielectric constant is 11.9. In other word, for the case discussed in our work, the substrate's relative dielectric constant has little effect on the extracted coating's conductivity.

5.1. Scanning Electron Microscope (SEM) Photo of the Deposited Aluminium Nano-Film

The following two graphs are SEM image of the first sample. It can be seen that the deposited aluminium nano-film is continuous and flat.

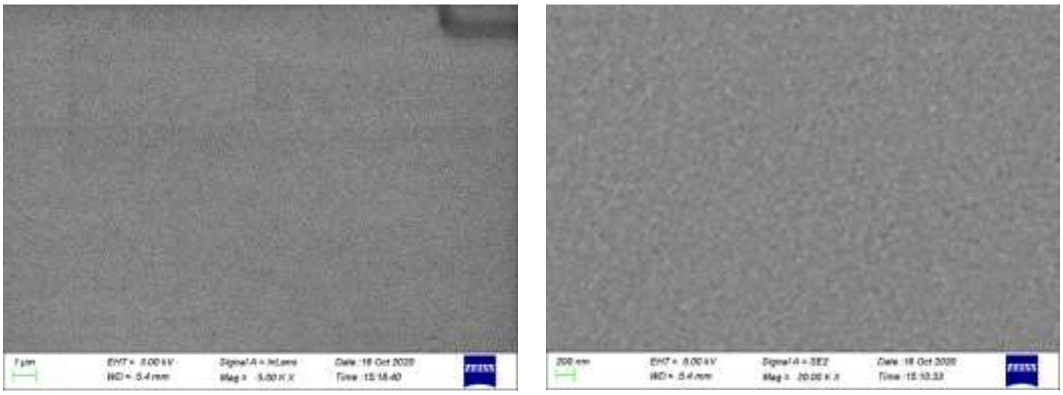

Figure S3. Scanning electron microscope (SEM) photo of deposited aluminium film

Publisher's Note: MDPI stays neutral with regard to jurisdictional claims in published maps and institutional affiliations.

© 2020 by the authors. Submitted for possible open access publication under the terms and conditions of the Creative Commons Attribution (CC BY) license (http://creativecommons.org/licenses/by/4.0/).
